# PTEN and Other PtdIns(3,4,5)P_3_ Lipid Phosphatases in Breast Cancer

**DOI:** 10.3390/ijms21239189

**Published:** 2020-12-02

**Authors:** Mariah P. Csolle, Lisa M. Ooms, Antonella Papa, Christina A. Mitchell

**Affiliations:** Cancer Program, Department of Biochemistry and Molecular Biology, Monash Biomedicine Discovery Institute, Monash University, Clayton, VIC 3800, Australia; mariah.csolle@monash.edu (M.P.C.); lisa.ooms@monash.edu (L.M.O.); antonella.papa@monash.edu (A.P.)

**Keywords:** phosphoinositide 3-kinase (PI3K), AKT, inositol polyphosphate phosphatases, phosphatase tensin homolog deleted on chromosome 10 (PTEN), proline rich inositol polyphosphate 5-phosphatase (PIPP), Src homology 2-containing inositol phosphatase 2 (SHIP2), synaptojanin 2 (SYNJ2), breast cancer

## Abstract

The phosphoinositide 3-kinase (PI3K)/AKT signalling pathway is hyperactivated in ~70% of breast cancers. Class I PI3K generates PtdIns(3,4,5)P_3_ at the plasma membrane in response to growth factor stimulation, leading to AKT activation to drive cell proliferation, survival and migration. PTEN negatively regulates PI3K/AKT signalling by dephosphorylating PtdIns(3,4,5)P_3_ to form PtdIns(4,5)P_2_. PtdIns(3,4,5)P_3_ can also be hydrolysed by the inositol polyphosphate 5-phosphatases (5-phosphatases) to produce PtdIns(3,4)P_2_. Interestingly, while PTEN is a bona fide tumour suppressor and is frequently mutated/lost in breast cancer, 5-phosphatases such as PIPP, SHIP2 and SYNJ2, have demonstrated more diverse roles in regulating mammary tumourigenesis. Reduced *PIPP* expression is associated with triple negative breast cancers and reduced relapse-free and overall survival. Although *PIPP* depletion enhances AKT phosphorylation and supports tumour growth, this also inhibits cell migration and metastasis in vivo, in a breast cancer oncogene-driven murine model. Paradoxically, SHIP2 and SYNJ2 are increased in primary breast tumours, which correlates with invasive disease and reduced survival. SHIP2 or SYNJ2 overexpression promotes breast tumourigenesis via AKT-dependent and independent mechanisms. This review will discuss how PTEN, PIPP, SHIP2 and SYNJ2 distinctly regulate multiple functional targets, and the mechanisms by which dysregulation of these distinct phosphoinositide phosphatases differentially affect breast cancer progression.

## 1. Introduction

Breast cancer is the most common cancer affecting women and the second leading cause of cancer death [1]. Despite recent advances in targeted treatments for primary tumours, metastasis is observed in nearly one third of breast cancers and is the leading cause of breast cancer mortality [2]. Breast cancers are classified into four major tumour subtypes based on expression of growth factor and hormone receptors, including estrogen receptor (ER), progesterone receptor (PR), and epidermal growth factor receptor 2 (ErbB2/HER2); this classification contributes to therapy decisions and provides prognostic significance [3]. Luminal A is the most frequent subtype of breast cancer and is generally characterised by the expression of ER and PR, and an absence of HER2 amplification [4]. Luminal B breast cancers express ER and HER2, and have a more aggressive phenotype than luminal A, with significantly worse prognosis [4]. HER2 positive breast cancers are characterised by the amplification of the *HER2* gene [4]. Historically, HER2 positive breast cancers have been associated with poor prognosis, although the development of targeted anti-HER2 therapies (e.g., trastuzumab) in the last decade has significantly improved overall survival [5]. Additional subtypes of breast cancer include the basal-like and triple-negative breast cancers (TNBCs), which lack expression of ER, PR and HER2 [4]. Due to a current lack of targeted treatment options, women diagnosed with TNBC have reduced three-year relapse-free survival compared to ER-positive (ER+) breast cancer [6].

There are a number of intracellular signalling pathways implicated in the initiation and progression of breast cancer. The phosphoinositide 3-kinase (PI3K) signalling pathway is one of the most frequently hyper-activated signalling networks in human cancer and is altered in approximately 70% of breast cancers [7]. In recent years, significant efforts have been undertaken to pharmacologically inhibit components of the PI3K pathway for cancer treatment, with some success. In a randomised, phase 3 trial, alpelisib, a selective inhibitor of the p110α catalytic subunit of PI3K, encoded by the *PIK3CA* gene, in combination with the ER antagonist fulvestrant, prolonged progression-free survival for *PIK3CA*-mutated, ER+, HER2-negative, advanced breast cancers that had previously received endocrine therapy [8]. However, disease progression and cancer relapse still occurred in the cohort receiving alpelisib/fulvestrant [8]. In relapsed chronic lymphocytic leukemia (CLL), idelalisib, a selective small-molecule inhibitor of p110δ, an isoform of the Class 1 PI3K catalytic subunit that is highly expressed in lymphoid cells, in combination with the CD20 monoclonal antibody rituximab, significantly improved progression-free and overall survival [9]. Despite these promising results, PI3K inhibitors have only shown modest single-agent therapeutic efficacy, highlighting the need for a better understanding of how PI3K and phosphoinositide signalling is mechanistically regulated in cancer.

## 2. The Phosphoinositide-3-Kinase (PI3K) Signalling Pathway

PI3Ks are a family of lipid kinases which phosphorylate the 3′-hydroxyl group of the inositol head group of membrane-bound phosphoinositides, critical signalling molecules that regulate a range of cellular functions including cell proliferation, survival, cytoskeletal re-organisation and vesicular transport [10]. PI3Ks are divided into three distinct classes depending on their amino acid sequence homology, associated regulator subunits, substrate preference, and different cellular distribution [10].

Class I PI3Ks play a major role in breast cancer pathogenesis, therefore, in this review we will focus on the class I PI3K signalling pathways. In mammals, class I PI3Ks are divided into two subclasses. Class IA PI3Ks consist of heterodimers containing a p85 regulatory subunit and a p110 catalytic subunit. The p85 regulatory subunit maintains p110α in a low-activity state until it interacts with phosphorylated tyrosine residues on receptor tyrosine kinases (RTKs), following activation by growth factor binding [11]. Class IB PI3K heterodimers consist of a p110γ catalytic subunit and a p101 regulatory subunit and are activated downstream of G-protein-coupled receptors (GPCRs) [12].

Once activated, class I PI3Ks phosphorylate phosphatidylinositol 4,5-bisphosphate (PtdIns(4,5)P_2_) to transiently generate phosphatidylinositol 3,4,5-trisphosphate (PtdIns(3,4,5)P_3_). Proteins with pleckstrin homology (PH) domains such AKT and PDK1 bind to membrane bound PtdIns(3,4,5)P_3_ on the inner wall of the plasma membrane and on early endosomes [11,13]. Once bound to PtdIns(3,4,5)P_3_, AKT is phosphorylated by PDK1 at Thr308 and the mTORC2 complex at Ser473 and becomes fully active [11,14]. Activated AKT phosphorylates numerous cytosolic and nuclear targets including GSK3β, PRAS40, FOXO, mTORC1 and p27 to regulate cell metabolism, proliferation, survival, and migration [14].

### 2.1. AKT Isoforms

There are three AKT isoforms in mammals; AKT1, AKT2 and AKT3, each derived from separate genes mapping to different chromosomes [15]. AKT1 is expressed in most tissues and AKT2 is predominantly expressed in insulin-responsive cells, whereas AKT3 expression is limited to neurons and mouse testes [16,17,18]. Generation of murine knockout mouse models has demonstrated AKT isoform-specific roles. For example, complete loss of *Akt1* (*Akt1*^−/−^) causes growth deficiency and increased spontaneous apoptosis in the testes and thymus of mice; in contrast, *Akt2*^−/−^ mice demonstrate a defect in insulin sensitivity, while *Akt3*^−/−^ mice show a dramatic reduction in brain size [17,19,20]. Despite sharing some common effectors, several studies have revealed non-redundant functions of the AKT isoforms, not only due to their distinct tissue expression, but also via activation of isoform-specific downstream targets [15].

The AKT isoforms play distinct, non-redundant roles in breast cancer. Expression of constitutively active AKT1 or AKT2 in murine mammary epithelial cells was not sufficient to drive de novo tumourigenesis [21,22]. However, in oncogene-driven murine breast cancer models, constitutively active AKT1 enhanced primary tumour initiation and growth, but paradoxically inhibited metastasis [21,22,23]. In contrast, activated AKT2 had no effect on tumour initiation but promoted metastasis in murine breast cancer models [21]. Ablation of single AKT isoforms in oncogene-driven mouse models of breast cancer has revealed more complex roles for AKT1 and AKT2 in regulating mammary tumour progression. Germline deletion of *Akt1* inhibited tumour development but had contrasting effects on metastasis where reduced, as well as increased, lung metastases has been reported in MMTV-*neu* mice [24,25]. However, inducible systemic *Akt1* deletion after tumour initiation, but not cell-autonomous *Akt1* ablation, inhibited metastasis in HER2-enriched mouse models of breast cancer by impairing neutrophil mobilisation [26]. Cell-autonomous *Akt2* ablation inhibited primary tumour formation whereas germline or inducible systemic deletion of *Akt2* enhanced tumour development [25,26] via elevated circulating insulin levels leading to hyperactivation of AKT1 [26]. Germline and systemic *Akt2* ablation also showed opposing effects on mammary cancer metastasis as murine germline deletion impaired metastasis but systemic loss of *Akt2* enhanced metastasis [25,26]. *Akt3* ablation in *neu* or *PyMT* oncogene-driven breast cancer mouse models resulted in a mild inhibitory effect on tumour induction [25]. However, further work is required to understand the role of AKT3 in regulating breast tumourigenesis.

### 2.2. PI3K Pathway Regulation

Under normal physiological conditions, activation of the PI3K/AKT pathway is under tight control by inositol polyphosphate phosphatases. The phosphatase tensin homolog deleted on chromosome 10, PTEN, is an inositol polyphosphate 3-phosphatase that negatively regulates PI3K signalling by dephosphorylating PtdIns(3,4,5)P_3_ to form PtdIns(4,5)P_2_, and thereby switches off AKT signalling [27]. However, PtdIns(3,4,5)P_3_ is also hydrolysed by a family of enzymes known as the inositol polyphosphate 5-phosphatases (5-phosphatases), which remove the 5-position phosphate from the inositol head group to produce PtdIns(3,4)P_2_, a phosphoinositide that can also bind and activate PDK1 and AKT [27]. PtdIns(3,4)P_2_ is also dephosphorylated by PTEN or by the inositol polyphosphate 4-phosphatases (INPP4A and INPP4B) to form PtdIns(4)P and PtdIns(3)P, respectively, in a reaction that terminates PI3K/AKT signalling [27].

Activation of the PI3K pathway is observed in ~70% of breast cancers and leads to aberrant activation of downstream signalling molecules, resulting in uncontrolled tumour growth, metastasis and drug resistance. PI3K signalling activation can occur due to oncogenic activation of positive upstream regulators, including mutations in growth factor receptors such as epidermal growth factor receptor or *HER2*, or via mutations in the lipid kinase *PIK3CA* itself (reviewed in [28]), or mutations in PI3K effectors such as the serine/threonine kinase, *AKT* (reviewed in [16]). Alternatively, PI3K/AKT signalling dysregulation in breast cancer can occur via altered expression or inactivating mutations of PTEN, or altered expression of the 5-phosphatases. Mutation of 5-phosphatases in cancer is a rare event.

## 3. PTEN

PTEN is a dual specificity protein and lipid phosphatase located on chromosome 10q23. PTEN consists of an N-terminal catalytic domain linked to a C-terminal C2 domain that is responsible for binding to the lipid membrane, protein stability and enzymatic activation [29]. *PTEN* is one of the most frequently mutated tumour suppressor genes in human cancer. *PTEN* somatic mutations are detected in a number of cancers including glioblastoma, melanoma, breast, endometrial and prostate cancers [30,31]. In addition, germline *PTEN* mutations occur in individuals diagnosed with “PTEN hamartoma tumour syndrome (PHTS)”, a genetic condition associated with increased risk of developing breast and thyroid tumours, as well as skin and brain hamartoma [32,33]. In addition, loss of PTEN activity can occur via post-translational modifications, which can affect PTEN protein stability and/or cellular localisation. *PTEN* is lost in approximately 40% of breast cancers and its inactivation is generally associated with worse clinical outcomes and reduced response to targeted therapies [34,35,36,37,38].

The functional role of PTEN in tumour suppression has been extensively dissected through the generation of multiple mouse models. Complete loss of *Pten* expression in mice (*Pten*-null mice) results in severe developmental defects and is embryonically lethal by ~day nine of gestation [39]. Heterozygous loss of *Pten* (*Pten*^+/−^ mice) generates viable mice however, by five months of age, they begin developing de novo tumours in multiple tissues including in the prostate, colon, adrenal and mammary glands, recapitulating many features found in human tumours with partial loss of *PTEN* [40]. Approximately 50% of female *Pten*^+/−^ mice develop de novo mammary tumours from 30 weeks of age [41]. Consistent with the role that PTEN plays in downregulating PtdIns(3,4,5)P_3_, tumours arising from *Pten*^+/−^ mice exhibit constitutive AKT phosphorylation [41]. *Pten* knock-in (KI) mice harbouring cancer-associated and loss-of-function *Pten* mutations such as the *Pten^C124S/+^* or the *Pten^G129E/+^* mutation, are highly tumour prone and develop tumours in multiple tissues including in the thyroid, adrenal gland, gallbladder, prostate and mammary gland, similar to *Pten^+/−^* mice [42]. Importantly, tumours driven by these, as well as other loss-of-function *Pten* mutations, often present with more advanced malignant features and display increased levels of AKT activation compared to *Pten*^+/−^ tumours. Mechanistically, upon dimerisation, PTEN mutations inhibit the function of the remaining wild-type protein and promote tumourigenesis in a dominant negative manner [42,43,44].

Mice with complete *Pten* deletion in the mammary epithelium (MMTV-*Cre+*) display precocious mammary lobulo-alveolar development, excessive ductal branching, delayed involution, severely reduced apoptosis, and a high frequency of mammary tumour formation, as early as two months of age [45]. Notably, 67% of *Pten* hypermorphic mice (*Pten*^hy/+^), expressing 80% of normal levels of PTEN, develop sporadic mammary tumours, indicating that even subtle reductions in PTEN levels can predispose to mammary tumourigenesis [46]. PTEN also regulates breast cancer progression at the tumour-stromal interface, independent of its role in suppressing tumourigenesis in epithelial cells. In murine breast cancer models expressing mammary-specific ErbB2/neu oncogene, loss of *Pten* in stromal fibroblasts via the mesenchymal-specific *Fsp-Cre* transgene, accelerated initiation, progression and malignant transformation of neu oncogene-driven mammary epithelial tumours, associated with extracellular matrix (ECM) remodelling, innate immune cell infiltration, and increased angiogenesis [47].

Consistent with in vivo findings, targeted *PTEN* deletion in the non-tumourigenic MCF-10A human mammary epithelial cell line increased PtdIns(3,4,5)P_3_ and PtdIns(3,4)P_2_ levels, as well as AKT activation following EGF-stimulation [48]. Similarly, antisense mediated reduction of *PTEN* expression in the MCF-7 ER+ breast cancer cell line resulted in constitutive hyperphosphorylation of AKT [49]. Reduced expression of *PTEN* also altered the cell cycle leading to a higher S-phase fraction compared to control cells [49].

PI3K inhibitors have been used to treat cancer with PTEN alterations. In particular, *PTEN*-negative breast cancers and cells with reduced *PTEN* expression exhibited increased sensitivity to the anti-proliferative effects of the pan-PI3K inhibitor LY294002 [49]; however, this drug has no clinical application due to its dose limiting toxicity. New isoform specific PI3K inhibitors have been generated and their efficacy has been assessed in relation to the functional status of PI3K and PTEN. Interestingly, while the selective p110α inhibitor alpelisib (BYL719) generated a positive response in *PIK3CA* mutant breast cancer, the additional loss of *PTEN* induced reactivation of p110β signalling and caused resistance to this targeted therapy [37,50,51]. Combined inhibition of p110α and p110β effectively induced apoptosis in vitro and in vivo and maintained proliferative arrest in *PTEN*-deficient ER+ breast cancer cells [37,52].

Although *PTEN* deficiency is associated with AKT activation, opposing functions of the various AKT isoforms in the regulation of tumourigenesis have been described in a context of *PTEN* deficiency. In *Pten^+/−^* mice, *Akt1,* but not *Akt2*, deficiency dramatically inhibited the development of a range of tumours [53,54]. In *PTEN*-deficient prostate cancer models, downregulation of AKT2, but not AKT1, promoted regression of prostate cancer xenografts through upregulation of p21 and the pro-apoptotic protein Bax, and downregulation of insulin-like growth factor receptor-1 [55]. In vitro, downregulation of AKT2, but not AKT1 or AKT3, attenuated cell proliferation and 3D spheroid growth in *PTEN*-deficient prostate and breast cancer cells [55]. These results indicate that *PTEN* deficiency causes tumorigenesis in an AKT-dependent manner, involving the differential activation of distinct AKT isoforms.

### PTEN Protein Phosphatase Activity in Tumourigenesis

In addition to the lipid phosphatase activity, PTEN also functions as a protein phosphatase towards phospho-tyrosine, -serine and -threonine residues [56,57]. The PTEN phosphatase domain contains the signature CX_5_R motif present in the active sites of many dual specificity protein phosphatases [58]. Interestingly, while this motif is also present in the active site of the PtdIns(3,4)P_2_ 4-phosphatase, INPP4B, it is not present in 5-phosphatases such as PIPP, SHIP2 or SYNJ2, which contain a large 300 amino acid catalytic domain that resembles the apurinic/apyrimidinic base excision repair endonucleases [59,60]. PTEN can suppress cancer signalling by dephosphorylating protein targets such as the focal adhesion kinase (FAK), the tyrosine kinase PTK6 (BRK), as well as PTEN itself, all of which can regulate breast tumourigenesis [61,62,63]. However, the generation of *Pten* knock-in mice harbouring the *Pten^G129E^* mutation, with loss of PTEN lipid phosphatase function only, and the *Pten^C124S^* mutation, with loss of PTEN lipid and protein phosphatase activity, has shown that PTEN regulation of PtdIns(3,4,5)P_3_ levels is the only essential PTEN function during embryonic development and in tumour initiation [42]. In addition, loss of PTEN lipid phosphatase function (*Pten^G129E/+^*) in mice with the *Pik3ca^H1047R^* mutation, promoted rapid development of advanced-stage and invasive mammary tumours, but the additional loss of PTEN protein phosphatase activity did not further exacerbate the phenotype [64]. Rather, on a *Pik3ca*-mutant background, *Pten^C124S/+^* mutation sensitized tumour epithelial cells and mammary organoids to cell death induced by the glucocorticoid receptor (GR) [64]. These findings demonstrate that under suboptimal and stressful growing conditions, loss of PTEN protein function triggers a failsafe mechanism in a cell-autonomous manner, that can be exploited in combination therapies with AKT inhibitors for breast cancer treatment [64].

## 4. Inositol Polyphosphate 5-Phosphatases

The inositol polyphosphate 5-phosphatase (5-phosphatase) family comprises 10 mammalian members; inositol polyphosphate 5-phosphatase A (INPP5A); inositol polyphosphate 5-phosphatase B (INPP5B); Lowe oculocerebrorenal syndrome protein (OCRL); synaptojanin 1 (SYNJ1/INPP5G); synaptojanin 2 (SYNJ2/INPP5H); proline rich inositol polyphosphate 5-phosphatase (PIPP/INPP5J); skeletal muscle and kidney enriched inositol phosphatase (SKIP/INPP5K); Src homology 2 (SH2)-containing inositol phosphatase 1 and 2 (SHIP1/INPP5H and SHIP2/INPPL1); and inositol polyphosphate 5-phosphatase E (INPP5E).

The 5-phosphatases all share a conserved 300 amino acid phosphatase domain flanked by various protein-specific functional domains such as the SKICH domain (SKIP carboxy homology), SH2 domains, proline-rich domains, and the RhoGAP and CAAX motifs; these domains are critical for correct protein function and localisation, and allow for a diverse range of tissue-specific roles [27]. Many 5-phosphatases play significant roles in human development and despite the shared amino acid sequence identity in their catalytic domains and key catalytic residues, the various functional roles of the 5-phosphatases appear to be non-redundant. Mutations in different family members lead to specific and severe developmental disorders such as Lowe syndrome and Dent disease (*OCRL*), or MORM syndrome and Joubert syndrome (*INPP5E*) [65], and most recently identified Marinesco–Sjögren Syndrome (MSS) caused by mutations in *INPP5K* [66,67].

All 5-phosphatases dephosphorylate lipid-substrates such as the second-messenger PtdIns(3,4,5)P_3_, and PtdIns(4,5)P_2_ [68,69,70,71,72,73,74,75,76,77,78], except for INPP5A which dephosphorylates the 5-position phosphate from only the inositol phosphates Ins(1,4,5)P_3_ and Ins(1,3,4,5)P_4_ [79,80]. Phosphorylation of PtdIns(4,5)P_2_ to PtdIns(3,4,5)P_3_ by PI3K activates AKT and its oncogenic signalling. Therefore, it would be expected that the 5-phosphatases would always act as negative regulators of PtdIns(3,4,5)P_3_ and suppress PI3K/Akt signalling, as seen with PTEN. Consistent with this, reduced expression of some of the 5-phosphatases, such as PIPP, can promote tumour growth [81]. However, paradoxically, increased expression of the lipid phosphatases SHIP2 and SYNJ2 can have pro-tumourigenic effects in breast cancer, indicating more diverse roles for the 5-phosphatases [82,83] (Table 1). Some of the other 5-phosphatases have been reported to regulate tumourigenesis in other cancer types such as glioblastoma (INPP5K) and medulloblastoma (INPP5E), but have not been implicated in the pathogenesis of breast cancer to date. The conundrum of why not all PtdIns(3,4,5)P_3_ phosphatases have “tumour-suppressive” functions in breast cancer will be the focus of the remainder of this review.

## 5. The Role of PIPP in Breast Cancer Suppression

The proline rich inositol polyphosphate 5-phosphatase (PIPP/INPP5J) contains two proline rich domains at the N- and C- termini and a central 5-phosphatase domain followed by a SKICH domain that localises PIPP to membrane ruffles [77,91]. PIPP dephosphorylates PtdIns(3,4,5)P_3_ to PtdIns(3,4)P_2_ to suppress AKT activation and oppose oncogenic PI3K signalling [77,92].

The gene encoding *PIPP/INPP5J* is located on chromosome 22q12. Allelic loss of chromosome 22q occurs in ~30% of breast tumours and loss of heterozygosity of this region frequently occurs in breast cancers [93,94]. Interestingly, a study of 13,547 genes identified *PIPP* as one of the top 10 genes for predicting outcome in breast cancers [95]. *PIPP* mRNA expression is decreased in ER-negative and triple-negative breast cancers and lower *PIPP* expression is associated with reduced relapse-free and overall survival [81,96].

**Figure 1:**
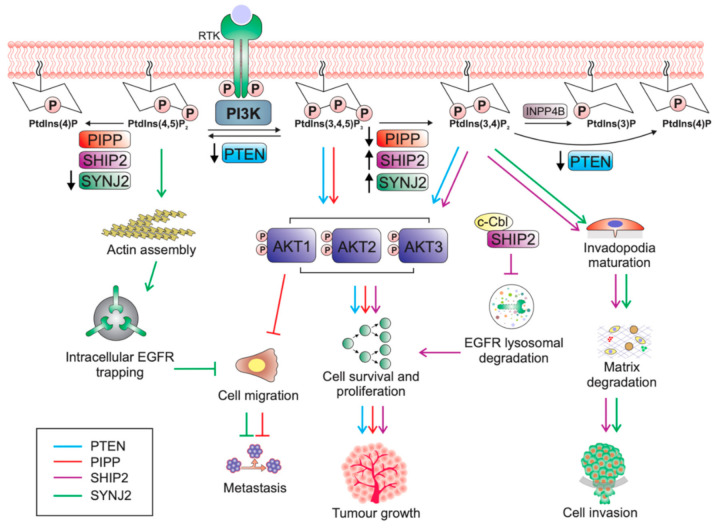
Pro- and anti-tumourigenic effects of PTEN and the 5-phosphatases in breast cancer. Class I PI3K phosphorylates PtdIns(4,5)P_2_ to form PtdIns(3,4,5)P_3_ at the plasma membrane in response to growth factor binding to a receptor tyrosine kinase (RTK). PtdIns(3,4,5)P_3_ facilitates activation of PH domain containing proteins such as AKT to regulate cell survival, proliferation and migration. PTEN negatively regulates PI3K/AKT signalling by dephosphorylating PtdIns(3,4,5)P_3_ and PtdIns(3,4)P_2_ to form PtdIns(4,5)P_2_ and PtdIns(4)P, respectively. Alternatively, PtdIns(3,4,5)P_3_ can be hydrolysed by the 5-phosphatases including PIPP, SHIP2 and SYNJ2 to produce PtdIns(3,4)P_2_, a phosphoinositide which also binds AKT. PtdIns(3,4)P_2_ is further dephosphorylated by the 4-phosphatases such as INPP4B to terminate AKT signalling. PTEN is a bona fide tumour suppressor and *PTEN* loss promotes mammary tumour initiation and growth through PtdIns(3,4,5)P_3_ and PtdIns(3,4)P_2_-mediated AKT activation (blue arrows). Similarly, PIPP is anti-tumourigenic and loss of *PIPP* increases PtdIns(3,4,5)P_3_ and AKT activation, enhancing cell survival and proliferation, and facilitating tumour growth (red arrows). Paradoxically, *PIPP* depletion inhibits cell migration and metastasis through AKT1 (red arrows). Despite being negative regulators of the PI3K/AKT signalling axis, SHIP2 and SYNJ2 are pro-tumourigenic. Increased *SHIP2* expression promotes cell survival, proliferation and tumour growth through PtdIns(3,4)P_2_-mediated AKT activation, as well as via inhibition of c-Cbl-mediated EGFR degradation (magenta arrows). SHIP2 and SYNJ2 also enhance tumour cell invasion by increasing localised PtdIns(3,4)P_2_ levels at the invadopodia core, promoting invadopodia maturation and matrix degradation (SHIP2 magenta arrows; SYNJ2 green arrows). *SYNJ2* depletion promotes PtdIns(4,5)P_2_-mediated accumulation of F-actin on intracellular vesicles, leading to impaired EGFR recycling and EGF-dependent migration (green arrows).

In cultured ER-negative MDA-MB-231 cells, shRNA-mediated knockdown of *PIPP* increased AKT phosphorylation following EGF stimulation and enhanced cell proliferation, survival and xenograft tumour formation. *Pipp* knockout mice are viable with normal mammary gland development and show no evidence of de novo mammary tumourigenesis [81]. However, *Pipp* ablation in the MMTV-*PyMT* oncogene-driven murine mammary cancer model resulted in enhanced mammary tumour initiation with accelerated growth of established tumours, concomitant with increased AKT activation [81]. Surprisingly, although *Pipp* ablation promoted tumour growth, *PyMT;Pipp*^−/−^ mice exhibited reduced numbers of lung metastases [81]. Importantly, *PIPP* depletion reduced cell migration and matrix degradation in an AKT1-dependent manner (Figure 1) via the downstream effectors TSC2, NFAT1 and MMP2. AKT1 exerts an inhibitory effect on breast cancer cell migration by destabilising TSC2, resulting in decreased activation of Rho [97] and also by promoting the degradation of the transcription factor NFAT1 in a GSK3β-dependent manner [98,99]. *PIPP*-depleted breast cancer cells showed decreased levels of MMP2 [81], which digests the basement membrane component collagen type IV to facilitate breast cancer cell invasion and metastasis [100,101]. MMP2 expression is regulated by AKT1 [102].

How PIPP regulates cell migration specifically via AKT1 is not known. There is no evidence of selective AKT isoform activation as phosphorylation of both AKT1 and AKT2 was enhanced in *PIPP*-depleted MDA-MB-231 cells following EGF stimulation [81]. Rather, the AKT1-dependent effects of PIPP on breast cancer cell migration may relate to the relative expression or intracellular localisation of the various AKT isoforms in mammary tumours and breast cancer cell lines. AKT1 expression is higher than AKT2 during the later stages of tumour development in oncogene-driven murine mammary cancer models [26,81]. However, AKT1 and AKT2 exhibit similar expression in MDA-MB-231 cells where *PIPP* shRNA knockdown also impaired cell migration and reduced expression of AKT1 downstream effectors [81,103], suggesting that relative AKT isoform expression may not be the only mechanism by which PIPP regulates AKT1-dependent cell migration.

The AKT isoforms exhibit distinct subcellular localisations in human breast cancer cell lines and the distinct subcellular compartmentalisation of inositol polyphosphate phosphatases and AKT isoforms may play a role in determining signal specificity. AKT1 is mostly cytoplasmic whereas AKT2 localises to the mitochondria and the cytoplasm and AKT3 localises to the nucleus and the nuclear membrane [103]. PIPP localises to membrane ruffles, as well as the cytosol, where it may regulate localised pools of PtdIns(3,4,5)P_3_ in migrating cells, leading to isoform-specific activation of AKT, however this compartmentalisation of signalling has yet to be demonstrated, unlike for other kinases and phosphatases. For example, the class II PI3K isoform, PI3K-C2γ, produces a pool of PtdIns(3,4)P_2_ on early endosomes that selectively activates AKT2 but not AKT1 [104]. Similarly, the inositol polyphosphate 4-phosphatase, INPP4B, which shows an early endosome and cytosolic distribution, selectively inhibits AKT2 activation on early endosomes of *Pten*-deficient thyroid cancer cells and thereby suppresses cell proliferation and anchorage-independent growth [105,106].

### PIPP Functions as a Tumour Suppressor in Other Cancers

PIPP can also function as a tumour suppressor in melanoma and oesophageal cancer. In melanoma, *PIPP* is commonly downregulated through DNA copy number loss or histone hypo-acetylation [107]. Overexpression of PIPP in melanocytes reduced AKT activation, cell proliferation and survival in vitro and impaired melanoma growth in xenograft models [107]. Furthermore, approximately 1/3 of *PTEN*-null melanomas also display *PIPP* deficiency, suggesting that PIPP co-operates with PTEN to suppress PI3K signalling [107]. In vitro, co-knockdown of *PIPP* and *PTEN* resulted in enhanced AKT activation and enlarged anchorage-independent cell growth colonies, despite fewer colonies forming, compared with knockdown of *PIPP* or *PTEN*-alone, suggesting PIPP and PTEN have non-redundant inhibitory effects on PI3K/AKT signalling [107].

In oesophageal squamous cell carcinoma (ESCC) PIPP expression is reduced compared to adjacent non-tumour tissues, and this expression inversely correlated with clinical stage, tumour nodule-metastasis (TNM) classification and overall patient survival [108]. Overexpression of PIPP in ESCC cell lines decreased tumour size as well as phosphorylation of AKT in xenograft tumour models [108]. In vitro, PIPP overexpression significantly decreased cell proliferation, anchorage-independent cell growth and phosphorylation of AKT [108]. Furthermore, miR-508, a micro RNA which directly suppresses multiple phosphatases including PIPP, PTEN and INPP4A, constitutively activated PI3K/AKT signalling [108]. Overexpression of miR-508 promoted an aggressive phenotype of ESCC both in vitro and in vivo and high miR-508 levels correlated with poor survival and increased activation of PI3K/AKT signalling as detected in a large cohort of ESCC specimens [108]. Collectively these studies suggest PIPP directly suppresses PI3K/AKT signalling in several cancers and may co-operate with PTEN.

## 6. The Pro-Tumourigenic Role of SHIP2 in Breast Cancer

In contrast to the role that PTEN and PIPP play in suppressing breast cancer progression, the Src homology 2 (SH2) containing inositol 5-phosphatases, SHIP1 and SHIP2, show more diverse roles. SHIP1 and SHIP2 share ~38% sequence homology, with a similar domain structure comprising an N-terminal SH2 domain followed by a catalytic 5-phosphatase domain and proline-rich regions [109]. SHIP2 also contains a C-terminal sterile alpha motif (SAM) domain that mediates protein:protein interactions [110]. SHIP1 and SHIP2 differ considerably in tissue and cellular expression. SHIP1 is predominantly expressed in hematopoietic cells, whilst SHIP2 is expressed ubiquitously [109]. *Ship1*-null mice are viable and fertile, but have a significantly reduced lifespan due to chronic myeloid cell hyperplasia resulting in myeloid infiltration of vital organs, including the lungs [111,112]. SHIP1 suppresses tumour growth in hematopoietic cancers via its negative regulation of PI3K/AKT signalling by hydrolysis of PtdIns(3,4,5)P_3_ to PtdIns(3,4)P_2_ [113,114]. A missense mutation within the 5-phosphatase active site of SHIP1, resulting in a valine to glutamine substitution at codon 684 and reduced catalytic activity, has been identified in acute myeloid leukemia, although this appears to be a rare event [114]. Leukemia cells transiently expressing mutant *SHIP1^V684E^* showed enhanced AKT phosphorylation in response to IL-3 and were resistant to apoptosis [114], suggesting that SHIP1 can negatively regulate leukemia growth by suppression of AKT activation.

The use of a SHIP1 inhibitor, 3α-aminocholestane (3AC), in mice increased granulocyte production, however, interestingly this chemical inhibition did not trigger the myeloid-associated lung consolidation observed in *Ship1*-null mice [115]. This could be a consequence of the transient and reversible nature of chemical inhibition as opposed to *Ship1* genetic ablation, or perhaps the absence of SHIP1 protein may also alter its protein interactions, leading to altered signalling complexes, independent of its lipid phosphatase activity [115]. Interestingly, SHIP1 inhibition via 3AC treatment in human multiple myeloma cells was cytotoxic and decreased AKT activation in vitro. The cellular uptake of exogenous PtdIns(3,4)P_2_ improved cell survival in 3AC treated leukemia cells in a dose-dependent manner, indicating that SHIP1 can also support growth of hematopoietic cancers through increased PtdIns(3,4)P_2_ and downstream AKT activation [115]. Growing evidence also suggests that PtdIns(3,4)P_2_ is not only a transient by-product in the removal of PtdIns(3,4,5)P_3_ but acts as a signalling molecule leading to AKT activation, therefore mediating a potential “pro-proliferative” role for the 5-phosphatases. Some studies have reported AKT activity directly correlates with PtdIns(3,4)P_2_ levels in vivo [116]. Furthermore, AKT binds more strongly to PtdIns(3,4)P_2_, leading to greater activation of AKT than is observed from binding PtdIns(3,4,5)P_3_ alone [116,117].

SHIP2, a well-established regulator of insulin signalling and metabolism [88,118] has a proposed oncogenic role in breast cancer. However, overexpression of SHIP2 in mice has not been reported to lead to cancer predisposition or de novo tumour development. Transgenic overexpression of SHIP2 in mice increases body weight and reduces glucose tolerance, by inhibiting insulin-induced AKT phosphorylation [89]. Additionally, *Ship2*-null mice are highly resistant to high-fat diet-induced obesity and exhibit increased insulin sensitivity and glucose tolerance, through increased insulin-induced activation of AKT in liver and muscle [88]. Therefore, SHIP2 plays a role in negatively regulating AKT activation, glucose metabolism, and insulin signalling. However, SHIP2 levels are increased in ER-negative primary breast tumours compared to normal tissues, and SHIP2 expression positively correlates with invasive disease and reduced survival, supporting a pro-tumourigenic role for SHIP2 in breast cancer [82,119,120,121,122]. SHIP2 expression is also positively associated with expression of cell surface receptors such as EGFR, HER2 and VEGF [82,121].

SHIP2 may promote breast tumourigenesis, not only by increasing PtdIns(3,4)P_2_-induced AKT activation, but also via its role in regulating epidermal growth factor receptor (EGFR) turnover (Figure 1). In MDA-MB-231 triple negative breast cancer cells SHIP2 is highly expressed and constitutively associated with c-Cbl ubiquitin ligase [119]. c-Cbl is required for ubiquitination of EGFR, which is critical for ligand-dependent internalisation and subsequent lysosomal sorting of the receptor [123]. The SHIP2-Cbl interaction in MDA-MB-231 breast cancer cells prevented c-Cbl ubiquitination of EGFR, thereby suppressing ligand-induced EGFR degradation [119,123]. Similarly, shRNA-mediated *SHIP2* silencing decreased MDA-MB-231 cell proliferation and reduced total EGFR protein levels via enhanced EGF-induced receptor degradation, which were both rescued upon re-expression of wild-type *SHIP2* [119]. Downregulation of *SHIP2* sensitised cells to EGFR inhibitors and resulted in increased apoptosis upon treatment with the EGFR inhibitors PD153035 and AG1478, compared to control cells, demonstrating a possible therapeutic option for *SHIP2* silencing in breast cancer [119].

In vivo, shRNA-mediated *SHIP2* silencing in MDA-MB-231 cells significantly delayed tumour growth, decreased tumour size, and reduced lung metastasis in xenograft tumour models in nude mice [119,124]. Furthermore, *SHIP2* silencing in MDA-MB-231 cells reduced EGF-induced AKT activation, cell adhesion and migration [125]. *SHIP2* silencing also significantly reduced the protein levels of the cytokine receptor CXCR4, an important determinant of metastasis which is activated downstream of EGFR-AKT signalling, indicating that the pro-tumourigenic role of SHIP2 may be mediated by EGFR-induced AKT activation [125]. Whether SHIP2 exerts its pro-tumorigenic effects through distinct AKT isoform-specific functions remains to be investigated.

An alternative mechanism proposes that *SHIP2* downregulation reduces MDA-MB-231 cell migration and metastasis through impairment of invadopodia formation, a process which requires the local production of PtdIns(3,4)P_2_ [124]. Invadopodia are actin-based protrusions uniquely found in invasive cancer cells, which enhance extracellular matrix degradation thereby promoting tumour invasion and metastasis [126]. Invadopodia formation requires enrichment of PI3K-produced PtdIns(3,4,5)P_3_ around the precursor core, followed by conversion of PtdIns(3,4,5)P_3_ to PtdIns(3,4)P_2_, the latter lipid recruits invadopodia-specific proteins, such as TKS5, to the plasma membrane during invadopodia maturation [85,127]. SHIP2 mediates the spatiotemporal conversion of PtdIns(3,4,5)P_3_ to PtdIns(3,4)P_2_, supported by its localisation at the invadopodium-core following PtdIns(3,4,5)P_3_ enrichment [85]. PI3K activity promoted the formation and activity of invadapodia, which was increased by overexpression of SHIP2 (Figure 1) [127]. Notably, SHIP2 inhibition decreased the formation of mature invadapodia and matrix degradation, however, did not affect precursor formation [85], consistent with its role in dephosphorylating PtdIns(3,4,5)P_3_ to PtdIns(3,4)P_2_.

An independent study further demonstrated that *SHIP2* depletion in MDA-MB-231 cells significantly reduced the number of proteolytically active invadopodia and overall matrix degradation capacity [128]. Rescue of both invadopodia formation and ECM degradation was observed in cells with re-expression of wild-type, but not catalytically inactive *SHIP2* [128]. Similarly, a decrease in the ability to rescue invadopodia formation and matrix degradation was observed in cells expressing SH2-mutant *SHIP2*, suggesting that SHIP2 recruitment to invadopodia may be mediated by its SH2 domain [128]. Surprisingly, re-expression of a *SHIP2* mutant that retained 5-phosphatase activity and an intact SH2 domain, but lacked the C-terminal proline-rich domain, also showed decreased ability to rescue invadopodia formation and matrix degradation. This mutant SHIP2 failed to recruit Mena, an actin filamin elongation factor to invadopodia sites, leading to delayed elongation of actin filaments and defective assembly of the actin network within invadopodia precursors [128]. These results indicate that in addition to its role in generating local PtdIns(3,4)P_2_, SHIP2 may promote invadopodia maturation in breast cancer cells through a scaffolding function.

### The Negative Role SHIP2 May Play in Cancer Progression

In contrast to its pro-tumourigenic role in breast cancer, SHIP2 negatively regulates cancer cell progression in gastric cancer cell lines. In gastric tumours SHIP2 is commonly downregulated compared with normal gastric mucosa [129]. SHIP2 overexpression in gastric cancer cells inhibited cell proliferation, induced apoptosis and suppressed cell migration and invasion [129]. In vivo, SHIP2 overexpression in SGC-7901 gastric cancer cells inhibited xenograft tumour formation in nude mice, by decreasing AKT activation, resulting in upregulation of p21, p27 and the pro-apoptotic protein Bim [129]. In contrast to breast cancer models, restoring AKT activation in gastric cancer cells rescued the inhibitory effect of SHIP2 on cell proliferation [129]. These findings suggest that the pro- or anti-tumourigenic effect of SHIP2, and perhaps of the other 5-phosphatases, is largely context specific. It is likely that in gastric cancer, the loss of SHIP2 PtdIns(3,4,5)P_3_ 5-phosphatase activity leads to enhanced AKT activation, resulting in increased cell proliferation and tumour growth, as observed with PIPP in breast cancer. Further studies should also investigate whether SHIP2 regulates EGFR turnover in other cancer types, such as gastric cancer.

Similar to its role in gastric cancer, SHIP2 also negatively regulates growth of glioblastoma cells. Overexpression of SHIP2 in the PTEN-deficient U87-MG glioblastoma cell line reduced PtdIns(3,4,5)P_3_ and AKT phosphorylation, despite PtdIns(3,4)P_2_ remaining high [75]. SHIP2 overexpression initiated cell cycle arrest in cultured glioblastoma cell lines by preventing the downregulation of p27, which is required for G1/S transition [75]. Further studies in the *PTEN*-null 1321 N1 glioblastoma cell line showed that shRNA-mediated *SHIP2* depletion increased glioblastoma cell migration [130]. Interestingly, cell migration was not affected by the addition of PI3K inhibitors in either control or *SHIP2*-depleted 1321 N1 cells [130]. This suggests that in these cells, cell migration is regulated independent of PI3K signalling, and instead may be regulated by the PtdIns(4,5)P_2_ 5-phosphatase activity of SHIP2. SHIP2 colocalises with PtdIns(4,5)P_2_ at the plasma membrane and *SHIP2* depletion increased PtdIns(4,5)P_2_ levels in *PTEN*-null N1 cells [130]. Overexpression of a PtdIns(4,5)P_2_ specific probe, which blocks PtdIns(4,5)P_2_ signalling in *SHIP2*-depleted cells, reduced cell velocity to a greater extent than observed by blocking PtdIns(3,4,5)P_3_ [130]. In addition, focal adhesion proteins FAK, paxillin and vinculin, which act downstream of PtdIns(4,5)P_2_, were upregulated in *SHIP2* depleted cells [130]. These results suggest that SHIP2 regulates cell migration through hydrolysis of PtdIns(4,5)P_2_ in *PTEN*-null glioblastoma cells. However, in a *PTEN*-positive glioblastoma cell line, LN229, shRNA-mediated depletion of *SHIP2* inhibited cell migration and PI3K inhibitors reduced cell velocity in both control and *SHIP2*-depleted cells [130]. This suggests that in the absence of PTEN where both PtdIns(3,4,5)P_3_ and PtdIns(3,4)P_2_ levels are increased, SHIP2 inhibits cell migration through hydrolysis of PtdIns(4,5)P_2_, whereas in PTEN expressing cells, SHIP2 inhibits cell migration through hydrolysis of PtdIns(3,4,5)P_3_ to PtdIns(3,4)P_2_. Importantly, this highlights how the 5-phosphatases can regulate tumorigenesis through different mechanisms, and these effects may also depend on the relative expression pattern of other lipid phosphatases, such as PTEN or INPP4B, or the relative levels of other phosphoinositide species. Further work is required to elucidate the downstream signalling that mediates these functions.

The role the 5-phosphatases play in regulating tumourigenesis may not only depend on their regulation of specific phosphoinositides, but also on their capacity to interact with other tissue-specific proteins. Independent of its lipid phosphatase activity, SHIP2 could also mediate its differential role in tumourigenesis via protein-protein interactions. SHIP2 interacts with various cancer related proteins such as c-cbl [131], p130Cas [87], filamin [86], vinexin [132], c-met [133], EphA2 receptor [134], RhoA [135] and c-Jun N-terminal kinase 1 (JNK)-interacting protein (JIP1) [136] by its N-terminal SH2 domain, C-terminal proline rich domain and unique SAM domain [129]. Therefore, SHIP2 may have a context-specific role in regulating cancer growth, depending on the tissue-specific expression of its interacting proteins as well as whether these protein-protein interactions are pro- or anti-tumourigenic.

## 7. SYNJ2 Promotes Breast Tumourigenesis

Similar to SHIP2, synaptojanin 2 (SYNJ2) has been identified as a putative oncogene in breast cancer. Two synaptojanin isoforms (1 and 2) play diverse roles in clathrin-mediated endocytosis [137,138]. SYNJ1 and SYNJ2 both have a similar domain structure containing an N-terminal Sac1 domain, a central 5-phosphatase domain and divergent C-terminal proline rich domains [139]. SYNJ1 is highly expressed in the brain, specifically in the nerve termini, whereas SYNJ2 is more widely expressed and strongly enriched at lamellipodia and invadopodia [140,141,142]. SYNJ1 has not been reported to have an association with cancer predisposition or progression. *Synj1*-null mice die shortly after birth and exhibit neurological defects accompanied by increased PtdIns(4,5)P_2_ and an accumulation of pre-synaptic clathrin-coated vesicles [137]. The phenotype of *Synj2* knockout mice has not yet been reported, however an N-ethyl-N-nitrosourea (ENU)-induced mutation in the catalytic domain of *Synj2* leads to progressive hearing loss due to cochleae degeneration and loss of cochlear hair cells in mice [90].

Although very few studies have investigated the role SYNJ2 plays in regulating breast cancer, one reported that copy number gain, or increased expression of *SYNJ2* in a small cohort of breast cancer patients correlated with shorter survival in ER+ tumours [83]. SYNJ2 expression is also associated with invasive disease, high tumour grade, cell proliferation and overexpression of HER2 [83]. *SYNJ2* depleted MDA-MB-231 cells exhibited a reduced ability to form tumours in xenograft models, which was restored upon re-expression of wild-type, but not catalytically-deficient mutant SYNJ2 [83]. Overexpression of SYNJ2 in MDA-MB-231 cells increased cell invasion in vitro, whereas shRNA-mediated knockdown markedly decreased the invasive ability of MDA-MB-231 cells [83]. Re-expression of wild-type but not catalytically-inactive SYNJ2 restored invasiveness, indicating that the catalytic activity of SYNJ2 is essential for cell invasion [83]. Similarly, mice injected with *SYNJ2* depleted cells or cells re-expressing catalytically-deficient mutant SYNJ2, showed a significant reduction in metastasis to the lungs [83].

Similar to SHIP2, SYNJ2 also plays a role in regulating EGFR turnover in breast cancer cells. In MDA-MB-231 triple negative breast cancer cells, EGFR localises to lamellipodia where it is required for EGF-induced cell migration [83]. Lamellipodia are actin-based protrusions found at the leading edge of the cell and are essential for cell motility. In shRNA-mediated *SYNJ2* depleted MDA-MB-231 cells, EGFR failed to localise to the lamellipodia, and instead accumulated in abnormal intracellular vesicles surrounded by F-actin [83], a phenotype rescued by re-expression of wild-type but not catalytically inactive SYNJ2 [83]. Abnormal vesicular accumulation of EGFR impaired EGFR recycling, as well as receptor sorting and degradation [83]. As endocytosis is dynamically regulated by actin remodelling, depletion of SYNJ2 most likely impairs the clearance of PtdIns(4,5)P_2_ on endosomes leading to reduced disassembly of PtdIns(4,5)P_2_-binding proteins, resulting in the accumulation of F-actin, and thereby intracellular trapping of EGFR [83]. Functionally, *SYNJ2* depleted MDA-MB-231 cells demonstrated decreased ability to migrate towards an EGF-gradient (Figure 1) [83]. SYNJ2 overexpressing cells showed increased EGFR recycling leading to receptor stabilisation and more sustained AKT signalling [83]. As SHIP2 and SYNJ2 both contribute to EGFR turnover, future studies should investigate whether PIPP also regulates receptor trafficking, as another mechanism by which this 5-phosphatase differentially regulates breast cancer progression, especially as PIPP is also recruited to membrane ruffles.

SYNJ2 may also mediate breast cancer cell invasion and metastasis through its role in promoting invadopodia formation. Similar to SHIP2, SYNJ2 localises to invadopodia where it hydrolyses PI3K generated PtdIns(3,4,5)P_3_ to form PtdIns(3,4)P_2_ (Figure 1) [83]. *SYNJ2* depleted MDA-MB-231 triple negative breast cancer cells showed impaired invadopodia formation due to defective PtdIns(3,4)P_2_ localisation resulting in mislocalisation of invadopodia markers such as the PtdIns(3,4)P_2_-binding protein TKS5 [83]. The matrix metalloproteinase, MT1-MMP, also failed to localise to invadopodia in SYNJ2 depleted cells, instead accumulating in large, actin-decorated vesicles, resulting in decreased proteolytic ability [83]. Furthermore, small compound inhibition of SYNJ2 decreased the invasive ability of MDA-MB-231 cells, without inhibiting the related protein SYNJ1, suggesting that SYNJ2 may be a potential druggable target to block breast cancer metastasis [83].

Unlike SHIP2 and SYNJ2, PIPP has not been reported to localise to invadopodia. However, expression of the matrix metalloproteinase 2 (MMP2), a regulator of breast cancer cell invasion and metastasis generally enriched at the invadopodia, is decreased in *PyMT;Pipp^−/−^* tumours [81]. Further studies are needed to demonstrate whether PIPP regulates invadopodia formation similar to SHIP2 and SYNJ2. In COS-7 cells, PIPP localises to ruffling membranes mediated via its N-terminal and C-terminal proline rich regions [78].

## 8. The PtdIns(3,4,5)P_3_ Phosphatases in Breast Cancer: Oncogenes Versus Tumour Suppressors?

The PtdIns(3,4,5)P_3_ phosphatases play divergent roles in regulating normal cellular functions and tumourigenesis (Table 1). Loss of PTEN function in mice leads to the development of de novo mammary tumours, and its mutation in humans leads to tumour predisposition demonstrating that PTEN is a bona fide tumour suppressor. The 5-phosphatases degrade the same substrate as PTEN, PtdIns(3,4,5)P_3_, but by hydrolysing a distinct phospho-site on the inositol ring, generating a different lipid product, PtdIns(3,4)P_2_, these phosphatases play a more complex role in regulating breast cancer progression. For instance, deletion of *Pipp* alone does not result in the development of de novo tumours, unlike *Pten*-deficient mice. However, loss of *Pipp* in an oncogene-driven mouse model increases mammary tumour burden while decreasing the number of lung metastases. Conversely, SHIP2 loss or overexpression does not predispose mice to cancer development, suggesting that whilst the 5-phosphatase demonstrates pro-tumourigenic functions in breast cancer cell lines, it is not a bona fide oncogene. However, further studies in oncogene driven mouse models of breast cancer are required to elucidate its function as a driver of mammary tumourigenesis. *Synj2*-null or transgenic overexpressing mice have also not yet been reported and warrant further investigation in the context of breast cancer to determine whether SYNJ2 is oncogenic in vivo.

Genetic mutations could also contribute to the tumourigenic roles of the PtdIns(3,4,5)P_3_ phosphatases. In addition to genomic loss, *PTEN* missense and nonsense mutations occur in 5.49% of breast cancers, whereas mutations in the 5-phosphatases are less common: *PIPP* 0.62%, *SHIP2* 1.79% and *SYNJ2* 3.6% of breast cancers (COSMIC database, cancer.sanger.ac.uk) [143]. The functional roles of these mutations are not well defined and further work is required to determine what effect they have on phosphatase function, protein-protein interactions or subcellular localisation.

### 8.1. PtdIns(3,4)P_2_ Is An Activator of AKT Signalling

While it is clear that PTEN asserts its tumour suppressive role via the hydrolysis of PtdIns(3,4,5)P_3_ and PtdIns(3,4)P_2_, thereby controlling AKT phosphorylation, the 5-phosphatases play a more diverse modulatory role in regulating multiple phosphoinositide species, and AKT activation. It was originally speculated that as negative regulators of PI3K/AKT signalling, the 5-phosphatases should also inhibit tumourigenesis via dephosphorylation of PtdIns(3,4,5)P_3_ to form PtdIns(3,4)P_2_; however growing evidence suggests that PtdIns(3,4)P_2_ is also critical for AKT activation. Some studies have reported AKT activity directly correlates with PtdIns(3,4)P_2_ levels in vivo [116]. Furthermore, AKT binds more strongly to PtdIns(3,4)P_2_, leading to greater activation of AKT than is observed from binding PtdIns(3,4,5)P_3_ [116,117]. Another study reported that PtdIns(3,4,5)P_3_ and PtdIns(3,4)P_2_ have distinct roles in determining AKT phosphorylation and activity, whereby overall AKT activity, as a cytosolic enzyme, was dependent upon the levels of PtdIns(3,4)P_2_, and only membrane-associated AKT activity was dependent upon PtdIns(3,4,5)P_3_ [144]. This concept is referred to as the ‘Two PIP Hypothesis’ whereby both PtdIns(3,4,5)P_3_ and PtdIns(3,4)P_2_ are required to sustain malignant transformation in an AKT-dependent manner [145].

Considering this complex network, the role of the 5-phosphatases in regulating breast cancer may depend on the relative level of other phosphoinositides present in the cell, as well as the expression of other lipid phosphatases which may be able to compensate or act downstream of PtdIns(3,4)P_2_. In the presence of PTEN or INPP4B, PtdIns(3,4)P_2_ produced by the 5-phosphatases can be converted to PtdIns(4)P or PtdIns(3)P, respectively, in turn suppressing AKT signalling. However, tumours with loss of PTEN and/or INPP4B may accumulate phosphoinositides such as PtdIns(3,4)P_2_, which would lead to increased AKT activity. Indirectly, this would result in a pro-oncogenic role for the 5-phosphatases. Further work is required to understand this complex interplay and how it may lead to the different roles of the 5-phosphatases in regulating breast tumourigenesis.

### 8.2. PtdIns(4,5)P_2_ and Cancer

As well as acting as a precursor for PtdIns(3,4,5)P_3_, PtdIns(4,5)P_2_ is a preferred substrate for many 5-phosphatases, including SYNJ2 [70]. PtdIns(4,5)P_2_ is the most abundant phosphoinositide at the plasma membrane and is emerging as a key regulator of actin polymerisation, and thereby cell migration (reviewed in [146]). Thus, another mechanism by which the 5-phosphatases demonstrate differential roles in regulating tumourigenesis may be through preferential substrate specificity in different cell types.

As previously discussed, SYNJ2 regulates EGFR recycling in breast cancer cells and SHIP2 inhibits glioblastoma cell migration through the hydrolysis of PtdIns(4,5)P_2_ [83,130]. Another 5-phosphatase, INPP5K, highly expressed in the brain, heart, kidney and skeletal muscle, also regulates glioblastoma cell migration through dephosphorylation of PtdIns(4,5)P_2_ [147,148]. INPP5K contains an N-terminal catalytic 5-phosphatase domain that dephosphorylates both PtdIns(4,5)P_2_ and PtdIns(3,4,5)P_3_ but shows higher affinity for PtdIns(4,5)P_2_ in vitro [70], and a C-terminal SKICH domain critical for mediating its intracellular membrane localisation to membrane ruffles and the endoplasmic reticulum [91]. Mice heterozygous for a mutation in *Inpp5k* show increased AKT phosphorylation in skeletal muscle as well as increased insulin sensitivity and reduced diet-induced obesity [149]. Recently, causative *INPP5K* mutations have been identified in families with Marinesco–Sjögren Syndrome (MSS) in which affected individuals display congenital muscular dystrophy, cataracts and intellectual disability [66,67].

INPP5K can be found either upregulated or downregulated in glioblastomas, relative to normal brain tissue; and, increased *INPP5K* expression in glioblastoma is associated with improved long-term survival [147]. shRNA-mediated knockdown of *INPP5K* in PTEN-deficient U-87MG glioblastoma cells had no effect on tumour survival or proliferation, indicating that *INPP5K* depletion does not further enhance the amplified PI3K/AKT signals that occur as a consequence of PTEN loss [147]. However, PtdIns(3,4,5)P_3_ and PtdIns(4,5)P_2_, both substrates of INPP5K, co-operatively regulate cytoskeletal re-organisation and thereby cell migration. Knockdown of *INPP5K* impaired cell migration associated with significantly increased PtdIns(4,5)P_2_, and decreased phosphorylation of the actin-regulatory protein cofilin, a PtdIns(4,5)P_2_ binding protein [147]. Interestingly, overexpression of INPP5K also impaired cell migration due to an inability of cells to sustain lamellipodia formation, as well as reduced incorporation of the PtdIns(4,5)P_2_ binding protein talin, into focal adhesions where it promotes focal adhesion stability. These results indicate that INPP5K mediates cytoskeletal reorganisation and migration in PTEN-deficient glioblastoma cells with elevated PtdIns(3,4,5)P_3_, via tightly controlled regulation of PtdIns(4,5)P_2_. Considering PIPP, SHIP2 and SYNJ2 also dephosphorylate PtdIns(4,5)P_2_, the role of the 5-phosphatases in regulating PtdIns(4,5)P_2_-effector interactions in breast cancer cells should be considered as another mechanism by which these enzymes may differentially regulate cell migration and invasion.

## 9. Concluding Remarks

Despite great advances in defining the role phosphoinositide phosphatases play in breast cancer, additional work remains to fully elucidate the mechanisms underlying the divergent functions of these critical enzymes. Although some of the opposing roles played by 5-phosphatases can be explained by differences in receptor tyrosine kinase trafficking, subcellular localisation of phosphoinositide effectors, and compensatory expression patterns of other phosphoinositide-regulatory enzymes, how all these factors and players intersect and regulate each other is still not completely understood. Moreover, as detailed in this review, a critical target of many phospholipid species is the proto-oncogene AKT, a master regulator of multiple biological functions that amplifies phosphoinositide signalling output. However, given the wide range of other effectors regulated by PtdIns(3,4,5)P_3_ and additional phosphoinositide species, we predict that many more functional targets will be identified that will allow a better understanding of the molecular mechanisms driving breast cancer progression.

## Figures and Tables

**Table 1:** Divergent roles of the PtdIns(3,4,5)P_3_ inositol polyphosphate phosphatases in regulating breast tumourigenesis.

PtdIns(3,4,5)P_3_ Phosphatase	Substrate Specificity	Cellular Localisation	Role in Breast Cancer	Mouse Models
PTEN	PtdIns(3,4,5)P_3_ to PtdIns(4,5)P_2_; PtdIns(3,4)P_2_ to PtdIns(4)P [48]	Plasma membrane, nucleus, ER and mitochondrial-associated membranes [84]	Tumour suppressor	***Pten-null*** Severe developmental defects; embryonically lethal [39] ***Pten^+/−^* mice** *de novo* tumours in multiple tissues including prostate, skin, colon adrenal and mammary gland [40] **Mammary epithelial cell-specific deletion of *Pten*** Precocious lobulo-alveolar development; excessive ductal branching; high frequency of mammary tumour formation [45]
PIPP	PtdIns(3,4,5)P_3_ to PtdIns(3,4)P_2_; PtdIns(4,5)P_2_ to PtdIns(4)P [77,78]	Ruffling membranes and cytosol [78]	Anti-tumourigenic; pro-migratory	***Pipp*-null** Normal mammary gland development; no evidence of *de novo* mammary tumour formation [81] ***PyMT;Pipp^−/−^* mice** Enhanced mammary tumour formation; decreased number of lung metastases [81]
SHIP2	PtdIns(3,4,5)P_3_ to PtdIns(3,4)P_2_; PtdIns(4,5)P_2_ to PtdIns(4)P [75]	Invadopodia, focal contacts, lamellipodia, membrane ruffles [85,86,87]	Pro-tumourigenic	***Ship2*-null** Resistant to high-fat diet induced obesity; increased insulin sensitivity and glucose tolerance [88] **Transgenic overexpression** Increased body weight; reduced glucose tolerance [89]
SYNJ2	PtdIns(3,4,5)P_3_ to PtdIns(3,4)P_2_; PtdIns(4,5)P_2_ to PtdIns(4)P (higher specificity for PtdIns(4,5)P_2_) [70]	Invadopodia, lamellipodia, membrane ruffles [83]	Pro-tumourigenic	**ENU-induced mutation in catalytic domain** Loss of cochlear hair cells; cochlea degeneration [90]
